# The Regulation and Function of Lactate Dehydrogenase A: Therapeutic Potential in Brain Tumor

**DOI:** 10.1111/bpa.12299

**Published:** 2015-09-17

**Authors:** Cara J. Valvona, Helen L. Fillmore, Peter B. Nunn, Geoffrey J. Pilkington

**Affiliations:** ^1^ Cellular & Molecular Neuro‐oncology Research Group University of Portsmouth, School of Pharmacy & Biomedical Sciences Portsmouth UK

**Keywords:** aerobic glycolysis, brain tumor, lactate, LDHA, therapeutic target, tumor metabolism, Warburg

## Abstract

There are over 120 types of brain tumor and approximately 45% of primary brain tumors are gliomas, of which glioblastoma multiforme (GBM) is the most common and aggressive with a median survival rate of 14 months. Despite progress in our knowledge, current therapies are unable to effectively combat primary brain tumors and patient survival remains poor. Tumor metabolism is important to consider in therapeutic approaches and is the focus of numerous research investigations. Lactate dehydrogenase A (LDHA) is a cytosolic enzyme, predominantly involved in anaerobic and aerobic glycolysis (the Warburg effect); however, it has multiple additional functions in non‐neoplastic and neoplastic tissues, which are not commonly known or discussed. This review summarizes what is currently known about the function of LDHA and identifies areas that would benefit from further exploration. The current knowledge of the role of LDHA in the brain and its potential as a therapeutic target for brain tumors will also be highlighted. The Warburg effect appears to be universal in tumors, including primary brain tumors, and LDHA (because of its involvement with this process) has been identified as a potential therapeutic target. Currently, there are, however, no suitable LDHA inhibitors available for tumor therapies in the clinic.

## Lactate Dehydrogenase

**Figure 1:**
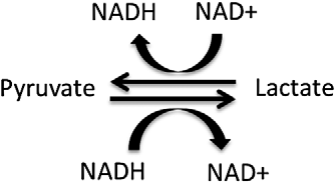
The reaction catalyzed by lactate dehydrogenase (LDH). LDH catalyzes the reversible conversion of pyruvate and NADH to lactate and NAD ^+^.

**Figure 2:**
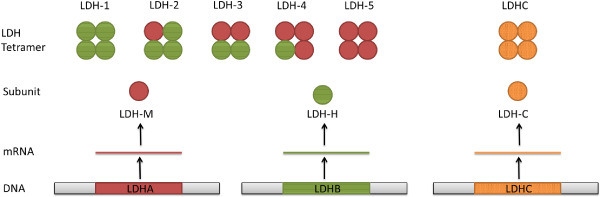
Lactate dehydrogenase (LDH) homo‐ and tetramer formation. The LDH isoenzymes LDH‐1, LDH‐2, LDH‐3, LDH‐4 and LDH‐5 are made up of different ratios of LDH‐M and LDH‐H subunits, transcribed from LDHA and LDHB, respectively. The LDHC tetramer is only made up of LDHC subunits.

Lactate dehydrogenase (LDH) is a tetrameric enzyme, belonging to the 2‐hydroxy acid oxidoreductase family, which increases the rate of the simultaneous inter‐conversion of pyruvate to lactate and nicotinamide adenine dinucleotide (NAD)H to NAD^+^ by 14 orders of magnitude 10 (Figure 1). The reaction involves the transfer of a hydride ion from NADH to the C2 carbon of pyruvate 99 and is commonly used by cells for anaerobic respiration. There are four LDH genes: *LDHA*, *LDHB*, *LDHC* and *LDHD* (Figure 2). *LDHA*, *LDHB* and *LDHC* are L isomers, whereas *LDHD* is a D isomer. The L isomers use or produce L‐lactate, which is the major enantiomer found in vertebrates.

The human *LDHA* gene is located on chromosome 11p15.4, the transcribed protein has 332 amino acids, a predicted molecular weight of 37 kDa and 24 splice variants; the human genome also contains several non‐transcribed LDHA pseudogenes 32, 126. Evolutionarily, *LDHA* and *LDHB* are thought to have arisen from the duplication of a single LDHA‐like LDH gene 82. *LDHC*, a testes‐specific gene, is also thought to have evolved in mammals from the duplication of the *LDHA* gene after the A‐B duplication 82.

LDHA is also known as the M subunit as it is predominantly found in skeletal muscle, and LDHB is also known as the H subunit as it is predominantly found in the heart. Unlike the other LDH genes, which can form only homotetramers, *LDHA* and *LDHB* can form homo‐ or heterotetramers. There are five isoenzymes of LDH that can be made from the M and H subunits: LDH‐1 (4H), LDH‐2 (3H, 1M), LDH‐3 (2H, 2M), LDH‐4 (1H, 3M), and LDH‐5 (5M) (Figure 2). LDH‐1 and LDH‐5 have identical active site regions and only differ in 81 out of 332 amino acid positions, most of which are found in the first 22 and last 38 residues and have a minimal effect on the overall structure 104. The N‐terminus of *LDHA* is important for structural stability as deletion of up to 10 amino acids from the N‐terminus increases instability, flexibility, inactivity and sensitivity to denaturing environments 150. Although structurally they are very similar, each LDH isoenzyme has different kinetic properties and studies suggest that their distinct kinetics are a result of the differences in charged surface residues bordering the active site 104. Each LDHA subunit has a net charge of −6 and a higher affinity for pyruvate, preferentially converting pyruvate to lactate and NADH to NAD^+^, whereas each LDHB subunit has a net charge of +1 and a higher affinity for lactate, preferentially converting lactate to pyruvate and NAD^+^ to NADH 61, 104.

**Figure 3:**
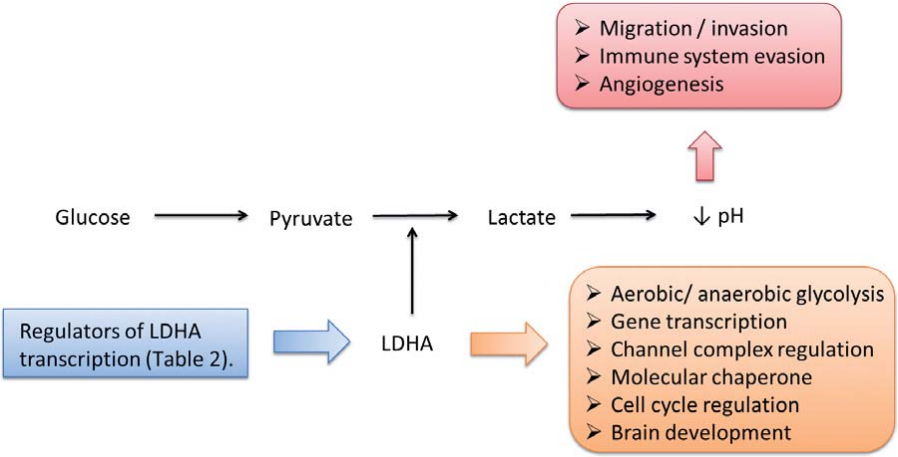
Schematic showing processes that are reportedly affected by lactate dehydrogenase A (LDHA). LDHA transcription is regulated by the genes and proteins listed in Table 2. LDHA has been reported to be involved with the processes listed in the orange box. LDHA has also been reported to indirectly influence the processes listed in the red box via aerobic glycolysis and lactate production.

*LDHA* has many roles in non‐neoplastic and neoplastic cells which are described in detail in the following text and summarized in Figure 3. The genes highlighted in the text, which have been reported to be associated with the function of LDHA, have also been compiled in Table 1. Most of the research into the function of LDHA has been demonstrated in non‐central nervous system (CNS) tumors, some of which commonly metastasize to the brain; however, links to primary CNS tumors will also be a focus in this review.

**Table 1:** List of genes and proteins reported to be associated with lactate dehydrogenase A (LDHA) activity

Gene/Protein	Association with LDHA function	Reference
AUF	Gene transcription	100
DNA polymerase‐α complex	Gene transcription	42
OCA‐S	Gene transcription	21, 43, 149
ATP‐sensitive K^+^ channel	Channel complex regulation	19
SUR2A	Channel complex regulation	19
Kir6.2	Channel complex regulation	19
NDPK‐A	Channel complex regulation	51
AMPK	Channel complex regulation	51
CK2	Channel complex regulation	51
IMT‐1	Molecular chaperone	34
Bax	Apoptosis	137, 144
PARP	Apoptosis	108, 137
Caspase 9	Apoptosis	113, 137
Caspase 3	Apoptosis	113
Bcl‐2	Apoptosis	108, 137
P53	Apoptosis	2
Bcl‐XL	Apoptosis	108, 153,
XIAP	Apoptosis	108
Mcl‐1	Apoptosis	153
Rcl	Tumor growth/survival	75
IDH	Tumor growth/survival	14
Oct‐4	Tumor growth/survival/cancer stem cell	145
MMP‐2	Tumor migration/metastasis	6, 113
E‐cadherin	Tumor migration/metastasis	113
FAK	Tumor migration/metastasis	113
THBS‐1	Tumor migration/metastasis	110
TGF‐β2	Tumor migration/metastasis	6, 110
Tropomyosin isoform (Tm5)	Tumor migration/metastasis	3
VEGF	Tumor angiogenesis	36, 54, 65, 113
VEGFR2	Tumor angiogenesis	36
NKG2D ligands: ULBP‐1 and MICB	Tumor evasion of immune response	17
Lactate	Transcription regulation of LDHA, tumor angiogenesis, tumor migration/metastasis, tumor evasion of immune response	6, 37, 69, 77, 101, 110, 125

## The Role of LDHA in Cellular Metabolism

**Figure 4:**
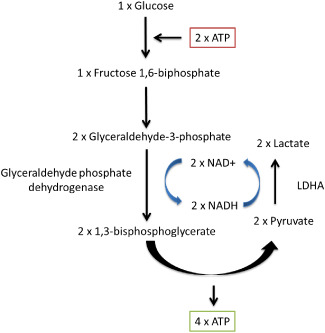
Anaerobic glycolysis. Lactate dehydrogenase A (LDHA) is required to maintain glycolysis and ATP production in the absence of sufficient oxygen by regenerating NAD ^+^ form NADH. Lactate is generated as the end by‐product of this reaction. The reaction consumes 2 ATP but creates 4 ATP, generating 2 net ATP per molecule of glucose.

Under normal physiological conditions, pyruvate is generated from glucose by glycolysis and enters the citric acid cycle in the mitochondria where it is oxidatively decarboxylated to form acetyl‐CoA, which is used to fuel oxidative phosphorylation, theoretically generating 36 net adenosine triphosphate (ATP) per molecule of glucose. However, when oxygen becomes scarce, cells are unable to use oxidative phosphorylation to efficiently generate ATP. In this scenario, glycolysis becomes the main generator of ATP, producing 2 net ATP per molecule of glucose. However, NAD^+^ is required to enable the sixth step of glycolysis as glyceraldehyde phosphate dehydrogenase (GAPDH) uses NAD^+^ to convert glyceraldehyde 3‐phosphate (GADP) to D‐1,3‐bisphosphoglycerate (1,3BPG). NAD^+^ is usually regenerated through oxidative phosphorylation by the electron transport chain, so when the oxygen supply is restricted, NAD^+^ is regenerated from NADH by LDHA in order to maintain glycolysis, generating lactate as a by‐product; this is known as anaerobic glycolysis (Figure 4). Although it is less efficient, anaerobic glycolysis is 100 times faster than oxidative phosphorylation, enabling it to fulfill the short‐term energy requirements in the absence of sufficient oxygen at the expense of a greater consumption of glucose.

Different tissues in the body have different metabolic rates, energy requirements and functions, which are often reflected in their LDHA : LDHB ratio. For example, approximately 40% of lactate in the circulation is released by skeletal muscle, whereas the liver and kidneys predominantly absorb lactate from the circulation and oxidize it to synthesize glucose 1. Brain metabolism is clearly complex as it responds dynamically to changes in blood glucose and lactate concentrations. A study of six normal physically active men found that at rest, the brain oxidizes approximately 10% of blood L‐lactate, fueling 8% of cerebral energy requirements, but still releases a small amount of net L‐lactate 129. However, in response to physical exertion and hyperlactatemia, the brain takes up net L‐lactate 94, 129, which contributes up to 60% of brain metabolism with cerebral lactate oxidation thought to use 33% of lactate 1, 94.

Cancer cell metabolism is modified when compared with that of normal cells and is known as the Warburg effect or aerobic glycolysis, first observed by Otto Warburg in the 1920s 138. Cancer cells use LDHA to elevate the rate of glycolysis, ATP and lactate production even when oxygen is available 55. Studies suggest that the switch to an aerobic glycolysis metabolic phenotype benefits cancer cells by avoiding generation of oxidative stress by the electron transport chain 68. Furthermore, by using aerobic glycolysis to generate ATP, cancer cells can use the intermediates of the citric acid cycle (which are regenerated by glucose and pyruvate) for anabolic reactions to synthesize the lipids, fatty acids and nucleotides required for rapid cell proliferation 22, 131. As discussed in previous reviews, this abnormal metabolism used by cancer cells is an attractive target for cancer‐specific therapies 97, 147.

## Regulation of LDHA Synthesis

The LDHA promoter region has long been known to contain the consensus sequences for, and be regulated by, major transcription factors: hypoxia‐inducible factor 1 (HIF1) and c‐Myc 29, 74, 111, 116. More recently, forkhead box protein M1 (FOXM1) 20 and Kruppel‐like factor 4 (KLF4) 114 were identified as transcriptional regulators of LDHA; however, LDHA regulation is complex and is far from being completely understood. LDHA transcription is also known to be influenced by many factors including lactate 77, cyclic adenosine monophosphate (cAMP) 85, estrogen 11, ErbB2 and heat shock factor 1 148 and is likely to be influenced by other unknown factors (Table 2).

**Table 2:** List of genes and proteins reported to regulate lactate dehydrogenase A (LDHA)

Gene/Protein	Mechanism of LDHA regulation	Reference
HIF1	Transcription regulation of LDHA	29, 111
C‐Myc	Transcription regulation of LDHA	74, 116
FOXM1	Transcription regulation of LDHA	20
KLF4	Transcription regulation of LDHA	114
cAMP	Transcription regulation of LDHA	85
Estrogen	Transcription regulation of LDHA	11
ErbB2	Transcription regulation of LDHA	148
Heat shock factor 1	Transcription regulation of LDHA	148
FGFR1	Post‐transcriptional regulation of LDHA	26
SIRT2	Post‐transcriptional regulation of LDHA	146

HIF1α is the alpha subunit of transcription factor HIF1 that is usually degraded under normoxic conditions by prolyl hydroxylase. However, under hypoxic conditions, HIF1α is stabilized and forms the HIF1 transcription factor with the constitutively expressed subunit HIF1β. HIF1 promotes the transcription of target genes involved in metabolism (including LDHA), angiogenesis and apoptosis, which support cell survival in hypoxic environments. However, HIF1α is also often stabilized in cancers, including brain tumors 105, by other factors such as overexpression of pyruvate kinase isozymes M2 (PKM2), Ras, proto‐oncogene tyrosine‐protein kinase Src and ErbB2 and constitutive activation of the phosphatidylinositol‐4,5‐bisphosphate 3‐kinase (PI3k), protein kinase B (Akt) or mammalian target of rapamycin (mTOR) pathway 79, 83. Furthermore, overexpression of HIF1α is associated with LDHA overexpression and a significantly poorer survival in some cancers 62, 78. Promotion of LDHA transcription by HIF1 has also been shown to be enhanced when cAMP binds to the cAMP response element (CRE) in the LDHA promoter region 29. However, LDHA regulation by HIF1 is clearly complex as studies have shown that LDHA creates a positive feedback loop, upregulating HIF1α expression under normoxic conditions by enhancing lactate production, which inhibits prolyl hydroxylase 77. On the other hand, studies in HT29 cells have shown that HIF1α expression was upregulated more in LDHA knockdown clones than control clones under hypoxic conditions, but unusually they also found that the upregulation of HIF1α in the LDHA‐silenced clones did not correlate with the expression of other HIF1‐regulated genes: carbonic anhydrase IX (CAIX) and vascular endothelial growth factor (VEGF) 70.

The c‐Myc proto‐oncogene is known as a “master regulator” as it regulates many key cell processes including cell cycle, growth, proliferation and apoptosis and is usually tightly controlled. For example, during exercise, c‐Myc expression and therefore LDHA expression are down‐regulated by peroxisome proliferator‐activated receptor‐γ co‐activator 1α (PGC‐1α) in oxidative muscle fibers to promote lactate uptake and lactate oxidation to maintain lactate homeostasis 122. However, c‐Myc expression is often deregulated in brain tumor cells, including the most metastatic subgroup of medulloblastoma (MB) (group 3) 124 and has been shown to transform rat fibroblasts by up‐regulating LDHA 74, 116. LDHA has also been shown to cooperate with Rcl, another c‐Myc target gene of unknown function, to induce anchorage‐independent cell growth *in vitro* and to induce tumor formation *in vivo* 75. The overexpression of c‐Myc can also enhance LDHA expression by promoting HIF1α stabilization under normoxic conditions and enhancing HIF1α expression under hypoxic conditions. Again, the regulation of LDHA by c‐Myc is not straightforward; studies in gastric cancer suggest that LDHA may be involved in a negative feedback loop, as inhibition of LDHA increases c‐Myc expression 145. Although HIF1 and c‐Myc have long been known to be LDHA transcription factors, there are still many aspects that are unclear and the precise mechanism in which they work and how their transcription of LDHA is regulated should continue to be actively investigated.

Recent studies have found that LDHA is also transcriptionally regulated by FOXM1 20. FOXM1 is an oncogenic transcription factor elevated in many types of cancer and is known to regulate the cell cycle, invasion, metastasis and angiogenesis 33, 60. Kim *et al* conducted studies that demonstrated that FOXM1 binds to the LDHA promoter region, upregulates LDHA mRNA transcription, LDHA protein expression, glucose consumption and lactate production 20. Furthermore, inhibition of LDHA activity diminished the enhanced glucose consumption and lactate production effects of FOXM1 overexpression, while *in vivo* studies established that upregulation of FOXM1 promoted LDHA expression, cancer growth and metastasis 20. Another recent study on tissue and cell lines found that LDHA expression was significantly positively correlated with pancreatic tumor progression and de‐differentiation, whereas KLF4, a transcription factor normally expressed in terminally differentiated epithelial cells, was significantly negatively correlated 114. Further examination found that KLF4 suppression significantly increased LDHA expression, whereas KLF4 overexpression significantly inhibited aerobic glycolysis, tumor growth and LDHA expression both *in vitro* and *in vivo* 114. Shi *et al* went on to find two KLF4 binding sites between −371 and −367 base pairs (bp), and −1310 and 1306 bp of the LDHA promoter region 114. These recent studies show promise and it would be interesting to see if the results are corroborated in different tissues, including brain tumors, as FOXM1 is a marker of poor prognosis in MB 102 and regulates glioma tumorigenicity 76. KLF4 is also suppressed in MB 89 and mutated in meningioma 106.

## Post‐Transcriptional Regulation of LDHA

Like many enzymes, LDHA post‐transcriptional activity is regulated by phosphorylation and acetylation of amino acid residues. The oncogenic receptor tyrosine kinase FGFR1, expressed in meningioma and glioma 128, has been shown to directly phosphorylate LDHA at Y10 and Y83 26. Y10 phosphorylation of LDHA, which is common in many human cancers, promotes active, tetrameric LDHA formation, whereas phosphorylation of Y83 promotes NADH substrate binding 26. Recently, Zhao *et al* have also shown that deacetylation of LDHA at lysine‐5 is regulated by SIRT2 deacetylase in pancreatic cancer 146. Furthermore, they found that the acetylation of LDHA at K5 leads to degradation of LDHA and proposed that it was caused by chaperone‐mediated autophagy (CMA) through interaction with HSC70 chaperone and lysosomes 146. Continued research on the mechanism of LDHA deactivation and degradation could aid in the development of novel therapeutic agents.

## The Role of LDHA in the Cell Cycle

It has been known since the 1960s that LDH expression fluctuates as cells progress through the cell cycle 56, 57. In 1991 Pan *et al* found that LDHA expression in normal human T and B lymphocytes increases when the cells are activated and proliferating, demonstrating that LDH isoenzymes can be used as proliferative markers 95. They also observed that LDHA expression intensity was at its greatest level when majority of T and B lymphocytes were in S/G2/M phase and that LDHA expression decreased as the cells returned to their resting state 95. LDH has since been used as a marker of cell proliferation and mobilization of CD34+ cells for stem cell apheresis 24, 25. More recent studies have begun to clarify the role of LDHA in the cell cycle. For example, inhibition of LDHA activity induced G2/M cell cycle arrest by downregulating the CDK1/cyclin B1 pathway in cell lines 144, while S‐phase transition was significantly induced by overexpression of LDHA 108. The mechanism by which LDHA affects the cell cycle warrants additional exploration.

## LDHA as a Transcription Factor

In the 1980s, it was reported that although LDHA is predominantly found in the cytoplasm, it is also localized in the nucleus of many tumors and binds to ssDNA and mRNA 41, 62, 63, 100, 112. Pioli *et al* showed that LDHA specifically binds to the AU‐rich element of GM‐CSF (granulocyte‐macrophage colony‐stimulating factor) RNA and interacts directly with AUF1 (a regulator of mRNA) 100. Furthermore, LDHA has been shown to upregulate DNA synthesis catalyzed by the DNA polymerase‐α complex up to fivefold 42 and bind translationally active RNA in polysomes 100. LDHA with tyrosine phosphorylation has also been reported as localized to the nucleus, suggesting that tyrosine phosphorylation may play an essential role in LDHA function in the nucleus 151. Together, these studies suggest that LDHA has a role in transcription, but the mechanism is still unclear and few specific targets have been identified. Although most of these studies were conducted over two decades ago, their importance must not be forgotten and should be investigated in more depth using modern technologies.

L‐LDH is critical in the organization of the Oct‐1 coactivator S (OCA‐S) transcription complex which regulates S‐phase histone 2B (H2B) transcription in a NADH/NAD^+^‐dependent manner 21, 43, 149. The OCA‐S complex was sensitive to cellular redox levels as H2B transcription decreased when NAD^+^ was depleted 21. Redox status and therefore metabolic status could be linked to gene switching, a mechanism that is commonly seen in prokaryotes and requires further exploration in relation to human DNA transcription. Collectively, these studies suggest one way in which LDHA could regulate transcription is through regulating the cellular redox state. However, these studies have not specifically identified whether LDHA or LDHB is involved predominantly or whether they have different enzymatic influences on the OCA‐S transcription complex.

## Other Roles of LDHA

LDHA has been shown to be an integral part of the sarcolemmal ATP‐sensitive K^+^ (KATP) channel in the heart, associating with the KATP channel subunits, SUR2A and Kir6.2, at the C‐terminus and N‐terminus, respectively. KATP channels are closed in response to high intracellular ATP but open during ischemia to prevent apoptosis. The generation of lactate by LDHA during ischemia allows the channel to open in the presence of ATP, protecting the cell from death caused by calcium accumulation 19. In this way, LDHA is able to couple KATP channel activity with the metabolic status of the cell and protect against cell death by ischemia. Interestingly, KATP channel expression is elevated in glioma and studies have shown that inhibition of KATP channels resulted in decreased glioma cell proliferation 47. The same group went on to discover that LDHA is part of the nucleoside diphosphate kinase‐A (NDPK‐A) isoform of the liver cytosolic substrate channeling complex 51. Furthermore, they found that LDH also has a role in regulating the activity of the liver cytosolic substrate channeling complex in response to the metabolic status of the cell. Knockdown of LDHA and LDHB revealed that LDHA upregulates the activity of AMPK and CK2, other components of the substrate‐channeling complex, whereas LDHB inhibits their activity 51. Together, these studies show how LDHA plays a critical role in the regulation of channel complexes in the heart and liver in response to the metabolic status of the cell. It is possible that LDHA could play similar roles in many other channel complexes. Other roles of LDHA also include acting as a molecular chaperone or as an association molecule during the differentiation of thymocytes 34.

## LDHA in the Brain and Cerebral Spinal Fluid

The brain is a very complex organ with high energy needs that change over a lifespan. Studies have shown that the human brain uses high levels of aerobic glycolysis during fetal growth and development, which increases during childhood, but then switches to oxidative phosphorylation which is seen predominantly in the adult brain 39. The ratios of LDHA to LDHB, which determine either aerobic glycolysis or oxidative phosphorylation, were examined in human fetuses at different stages of gestation. They found that LDHB expression decreased from 18% at 16 weeks to 15% at 40 weeks, compared to 23% in adult brains. LDHA increased from 8% at 16 weeks to 16% at 40 weeks, compared to 6% in adult brains 121. More recent studies have examined the distribution of LDHA and LDHB within various regions of the brain. Studies in mouse and rat brains showed that LDHB mRNA expression was predominant throughout the brain with the exception of strong LDHA expression in the hippocampal regions CA1, CA2 and CA4, the ventromedian hypothalamic nucleus, and the dorsal raphe nucleus as well as moderate expression in the cerebral cortex 71, 109. Moreover, a study of 33 neurologically normal young adults using magnetic resonance imaging (MRI) and positron emission tomography (PET) scans found that aerobic glycolysis was significantly elevated in a few regions, particularly the medial and lateral parietal and prefrontal cortices, whereas the cerebellum and medial temporal lobes had significantly lower levels of aerobic glycolysis than the rest of the brain 130. Recent studies have shown that the regions of the brain with the highest level of aerobic glycolysis also express increased levels of genes relating to synapse formation and growth, suggesting aerobic glycolysis could be used in brain development to support the biosynthesis required for growth, similarly to cancer cells 39. Others have also suggested that the varying levels of aerobic glycolysis within the human brain could be linked to the ratio of neurons to non‐neuronal cells 130. Aerobic glycolysis provides astrocytes with the high energy requirements of the membrane bound Na^+^/K^+^‐ATPase pump, at the cell surface, to take up glutamate from the synapse 98. According to the astrocyte–neuron lactate shuttle theory, the glucose metabolized to lactate by astrocytes is secreted and metabolized by neurons, which have no direct access to glucose 44. LDHA clearly has an active role within the brain and lactate has also recently been implicated as a neural intracellular messenger 5, but further research is required to determine its precise role.

Interestingly several mouse model studies have suggested that loss of aerobic glycolysis in the brain is linked to Alzheimer's disease. APP/PS1 (APPswe, PSEN1dE9) double transgenic mice are used as a model for Alzheimer's disease and studies showed that 12‐month‐old APP/PS1 mice had decreased PDK1 (pyruvate dehydrogenase kinase isozyme 1), a promoter of aerobic glycolysis, and LDHA expression in their frontal cortex compared with age‐matched controls 90. Furthermore, knockdown of LDHA or PDK1 in B12, a rat CNS cell line, increased their sensitivity to Aβ and other neurotoxins 90. Aβ deposition promotes dysfunction of mitochondria, ROS (reactive oxygen species) generation, and eventually leads to nerve cell death. By elevating PDK1 and LDHA expression and promoting aerobic glycolysis, the brain is protected 90. Furthermore, a neuroimaging study in humans found that the regions of the brain most susceptible to amyloid toxicity also exhibited high levels of aerobic glycolysis 133; this has been suggested to be a preventative protective measure against Aβ deposition and loss of this protective mechanism may lead to Alzheimer's disease 90. However, a study in prematurely and normally aging mice found failure of oxidative phosphorylation, thought to be caused mainly by mitochondrial DNA point mutations, and elevated brain lactate concentrations caused by increased LDHA transcription correlated with an aging phenotype 109.

Changes in the cerebral spinal fluid (CSF) LDH isoenzyme (Figure 2) concentrations can be used as an indicator of neurological pathologies. For example, normal CSF contains very low concentrations of LDH‐1 and LDH‐2 LDH isoenzymes, whereas LDH4 is elevated in tuberculous meningitis; LDH3 in pyogenic meningitis and Guillain–Barré syndrome; LDH2 in viral encephalitis and LDH1 in hydrocephalus 52, 92. Elevated CSF concentrations of LDH are also seen in patients with ischemic stroke and head injuries 96, 127. Furthermore, a study of leptomeningeal metastases from solid and hematological tumors found that CSF LDH concentrations could be used to help diagnosis and monitor the patient's response to treatment 127. There are several hypotheses for the cause of increased CSF LDH concentrations, including disruption of the blood brain barrier (BBB) which allows an increased outflow of serum, release of LDH from cytolytic cells, elevated synthesis of LDH in response to vascular damage or reduced removal of LDH 96. Further research is required to determine the mechanisms of CSF LDH upregulation and its reliability as a prognostic marker for brain tumors.

## LDHA and Tumor Malignancy

Abnormal LDHA upregulation and LDHB downregulation is a common characteristic of tumors, which promotes a metabolic switch to aerobic glycolysis, generating lactate as a by‐product. Elevated lactate concentrations have been shown to predict tumor malignancy, recurrence, survival and metastasis in cancer patients 9, 135, 136. Serum LDH concentrations have also been shown to be a good prognostic marker of many types of cancer 50, 78, 91, 139. LDHA overexpression is also associated with many other poor prognostic factors, including tumor hypoxia 111, angiogenesis 59, proliferation and glucose uptake 41, as well as resistance to chemotherapy 66 and radiotherapy 67. Furthermore, in non‐small‐cell lung cancer, which commonly metastasizes to the brain, LDHA expression and NF‐κβ are synergistically associated with death and recurrence 88. Several studies have also demonstrated that LDHA has a role in tumor formation, maintenance and progression 27, 72, 75; however, there has not been adequate research into the use of LDHA as a prognostic marker for brain tumors.

## The Role of LDHA in Tumor Growth and Survival

Several studies, including *in vivo* research, have attenuated LDHA expression initiated by various mechanisms and reports predominantly indicate that LDHA suppression inhibits tumor cell proliferation and survival 70, 72, 140, 145. However, Langhammer *et al* found that the growth of clones with LDHA silenced was not impeded *in vitro*, but the same LDHA‐silenced clones had an impaired growth rate *in vivo*, compared with the control cell lines 70. Additionally, cell lines that rely more on glutaminolysis and fatty acid synthesis are not affected by LDHA inhibition, whereas cell lines that rely on the pentose phosphate pathway and glycolysis are affected 8. Therefore, the importance of LDHA in cell growth and survival is likely to be dependent on tumor metabolic phenotype, reliance on LDHA and microenvironmental influences.

Predominantly, studies have found that LDHA indirectly promotes tumor survival through protection from ROS, as the inhibition of LDHA forces cells to use oxidative phosphorylation in order to generate ATP and mitochondrial ROS production is usually elevated as a result 27, 49, 72. Multiple *in vitro* and *in vivo* xenograft mouse model studies have found that LDHA knockdown cells treated with N‐acetyl‐L‐cysteine (NAC), an antioxidant that breaks disulfide bonds, prevented or partially prevented the induced generation of ROS and apoptosis 113, 137, 140. Furthermore, Sheng *et al* showed that the knockdown of LDHA and generation of ROS, which regulates the intracellular calcium concentration 103, caused a loss of mitochondrial membrane potential, release of cytochrome C, activation of caspase 9 and caspase 3, and finally apoptosis 113. *In vivo* xenografts of breast cancer cell lines also found that cell lines with LDHA knocked down had elevated Bax, cleaved PARP, cleaved caspase‐9, cytosolic cytochrome C and superoxide anion expression but decreased Bcl‐2 expression and mitochondrial membrane potential 137. One group has specifically looked at the effect of LDHA inhibition in p53+/+ and p53−/− tumors 134. They found that LDHA inhibition in both p53+/+ and p53−/− caused increased ROS and decreased ATP which lead to apoptosis, although p53+/+ cell lines were more sensitive to LDHA silencing, but no effect was seen on the viability of non‐neoplastic cell lines ARPE19 (retinal epithelia) and WI38 (diploid lung fibroblasts) 2. The increased sensitivity to LDHA inhibition in p53+/+ colorectal epithelial cancer cells was caused by a p53‐dependent increase in cellular NADH : NAD^+^ ratio, which resulted in downregulation of the activity of the p53 NAD^+^‐dependent deactylator SIRT1 and therefore upregulated acetylated, active tumor suppressor p53 2. Furthermore, LDHA suppression increased sensitivity of p53+/+ cancer cells to EO9, a redox‐dependent prodrug reduced by NADPH‐quinone oxidoreductase 1 (NQO1) 2.

LDHA may also inhibit apoptosis more directly. Indeed, an immunohistochemical study of melanoma by Zhuang *et al* revealed that not only did LDHA expression increase as the disease progressed but it was strongly associated with the expression of the anti‐apoptotic proteins Mcl‐1 and Bcl‐XL 153. Furthermore, knockdown of LDHA has been shown to increase PARP expression, decrease XIAP, Bcl‐2 and Bcl‐XL expression, and attenuate the tumorigenicity of the pancreatic cell line BXPC‐3, reducing the tumor size and weight *in vivo* xenograft models 108.

Interestingly, recent studies have demonstrated that LDHA is inhibited in the isocitrate dehydrogenase (IDH) subgroup of glioma, which has characteristically slow progression, greater survival rates and better prognosis than the other glioblastoma multiforme (GBM) subgroups 14. It has previously been shown that 2‐hydroxyglutarate (2‐HG), produced by IDH mutant tumors, promotes HIF1α degradation 58. Further *in vitro* and *in vivo* xenograft studies by Chesnelong *et al* established that LDHA, a HIF1α responsive gene, was underexpressed in different grades of IDH mutated gliomas 14. Even brain tumor stem cell (BTSC) lines that once had IDH mutations but lost their mutant IDH allele and no longer produced 2‐HG had silenced LDHA. These results led to the discovery that the LDHA promoter was heavily methylated 14. Furthermore, addition of mutant IDH to human astrocyte cell lines was also associated with methylation of LDHA promoter. To corroborate their findings, they analyzed data from The Cancer Genome Atlas and REMBRANDT public databases; they found that low expression of LDHA and high methylation of the LDHA promoter was found in IDHmt glioblastoma (GBM) patients and glioma patients whose tumors overexpressed LDHA had a median survival of 16 months, whereas patients whose tumor underexpressed LDHA had a median survival of >50 months 14. As LDHA has previously been shown to be important in tumor growth and progression in many other tumors, the silencing of LDHA in gliomas with IDH mutations may be responsible in part for the characteristically slow progression of gliomas with IDH mutations. These findings should prompt further studies to establish if LDHA correlates with tumor growth in other types of brain tumor.

There are several other possible mechanisms in which LDHA may promote tumor growth. LDHA may be involved in promoting the cancer stem cell phenotype; Zhang *et al* showed that LDHA significantly correlates with Oct‐4, a gene involved with embryonic stem cell self‐renewal 145. They conducted *in vitro* and *in vivo* studies using lentivirus‐mediated siRNA against LDHA and found that it reduced Oct‐4 expression and tumorigenicity 145. LDHA overexpression may also promote tumor growth by preventing necrosis in hypoxic environments; Lewis *et al* noted that tumors created from cell lines overexpressing c‐Myc or c‐Myc target genes LDHA and Rcl were not significantly necrotic compared with tumors from cell lines overexpressing c‐Myc target genes Rcl and VEGF 75. These observations show promise and should be investigated in both primary and secondary brain malignancies.

## The Role of LDHA in Tumor Migration and Metastasis

Secondary brain metastases are the most common type of adult brain tumor and the incidence is rising. Many cancers can metastasize to the brain but the most common are melanoma, breast, lung, kidney and colon cancer. LDHA expression correlates with metastasis and poor patient prognosis in many tumors 9, 41, 48, 50, 64, 153. The most frequently reported mechanism by which LDHA modulates cell migration and invasion is through the generation of lactate, as lactate alone has been shown to correlate with the invasive ability of many tumors 140. Lactate causes acidification of the microenvironment which promotes tumor cell invasion by inducing apoptosis of normal cells and pH‐dependent activation of metalloproteinases (MMPs) and cathepsins which degrade the extracellular matrix and basement membranes 6, 53, 107, 123. Goetze and others have used Transwell^®^ Boyden chamber assays to show that addition of exogenous lactate increased migration tumors in a concentration‐dependent manner 37. Sheng and others also showed that knockdown of LDHA reduced the expression of MMP‐2 and metastatic potential, using cell lines and xenograft mouse models 113. Furthermore, they found that the knockdown of LDHA caused an increase in the tumor suppressor E‐cadherin and therefore cell–cell adhesion, and a loss of focal adhesion kinase (FAK) and VEGF, both of which are also associated with tumor metastasis, considerably implicating LDHA as a regulator of invasion 113. There have been a few studies of LDHA and lactate in high‐grade glioma migration. Seliger *et al* found that the knockdown of LDHA resulted in a decrease in lactate concentration, which caused a reduction of THBS‐1 and TGF‐β2 expression and reduced migration by approximately 40% compared with the control siRNA 110. Furthermore, addition of lactate or synthetic THBS‐1 rescued TGF‐β2 expression and glioma migration 110. In another study, it was found that MMP‐2, a promoter of migration and invasion which is overexpressed in high‐grade glioma, is also upregulated by LDHA through lactate induction of TGF‐β2 6.

Arseneault *et al* reported another mechanism in which LDHA may regulate cell migration. They found that elevated mitochondrial ROS production, caused by LDHA‐targeted knockdown using shRNA, is associated with compromised actin dynamics, oxidation of tropomyosin isoform Tm5 and decreased cell motility in the melanoma‐derived cell line MDA‐MB‐435 3. In wound healing and transwell migration assays, migration of clonal MDA‐MB‐435 cell lines with knocked down LDHA was considerably decreased. Furthermore, addition of antioxidant NAC increased migration of a LDHA knockdown cell line in a concentration‐dependent manner. Together, their studies suggest that LDHA may influence cell migration through mitochondrial ROS production, Tms and redox regulation 3. Collectively, these studies suggest that LDHA‐targeted therapy could reduce tumor invasion and migration, which severely decreases a patient's chance of survival, especially in the context of primary brain tumor.

## The Role of LDHA in Angiogenesis

Angiogenesis is a hallmark of many tumors, including GBMs, and is stimulated by angiogenic factors including VEGF and IL‐8. Koukourakis *et al* published a number of immunohistochemical studies showing an association between LDHA expression and activation of the VEGF pathway 36, 59, 62, 65, 66. They found that a high level of LDHA in the cytoplasm correlated significantly with cytoplasmic VEGF expression 62, while LDHA expression was significantly associated with phosphorylated VEGFR2 in both tumor‐associated vasculature and tumor cells 36. Another group used a tissue microarray and found that high LDHA and VEGF expression in tumor and stroma was a prognostic factor for gastric tumors 54. However, these associations could be partly due to VEGF and other angiogenic factors also being a target of the LDHA transcription factors, HIF1 and c‐Myc. Furthermore, other immunohistochemical studies by Koukourakis *et al* found a significant correlation between LDHA, HIF1α and an aggressive phenotype. However, there was no association found between LDHA expression and VEGF or vascular density 64, 65.

Interestingly, an acute acidic extracellular pH, which can be caused by elevated lactate production, has been shown to promote upregulation of IL‐8 and VEGF independently of hypoxia 7, 35, 45, 115, 142. Studies in wound healing mouse models have shown that sustained, local and systemic lactate release from subcutaneous implants of poly‐D,L‐lactide‐co‐glycolide (PGLA) promotes angiogenesis and accelerates wound closure 101. A study where Hunt–Schilling wound cylinders were implanted into rats established that increased extracellular lactate concentrations in wounds caused an elevation of interleukin‐1β, VEGF (twofold) and TGF‐β1 (two‐ to threefold), a 50% increase in collagen deposition and a 90% reduction of insulin‐like growth factor‐1 125. Another study in HUVEC (human umbilical vein endothelial cells) cells cultured with 10 mM lactate found that VEGF and VEGFR2 were significantly upregulated 69. Furthermore, the VEGF expressed in the presence of lactate was of a more active form, as VEGF PAR‐modification decreased from 76% in the control to 21% in lactate‐treated cells 69. Another group developed a novel *in vivo* microscopy technique in order to simultaneously measure tissue oxygen partial pressure (pO_2_), pH and VEGF promoter activity in human glioma cells 35. They found that VEGF expression was independently regulated by pH and tissue pO_2_. Furthermore, hypoxia and acidic pH did not have a synergistic effect on VEGF transcription 35.

LDH concentrations may also be useful to predict whether patients will benefit from VEGF targeted therapies 13. A study of tissues from 179 colorectal cancer patients showed that patients treated with an anti‐angiogenic drug (Vatalanib) and cytotoxic chemotherapy [oxaliplatin/5‐fluorouracil (FOLFOX) (Bayer Schering Pharma AG, Berlin; Novartis, East Hanover, NJ, USA)] combination had an improved response rate and progression‐free survival if their tumor expressed elevated LDHA compared to patients with tumors expressing low levels of LDHA 66. Furthermore, a clinical trial of the anti‐angiogenesis drugs bevacizumab and cediranib on advanced colorectal cancer patients established that patients with high concentrations of serum LDHA (treated with cediranib) had better overall survival (OS), whereas patients with low concentrations of serum LDHA (treated with bevacizumab) had a better OS, although these results were not significant 4. Several studies suggest that LDHA could be involved in the promotion of angiogenesis, but further research is required to determine the exact mechanism that could then be targeted for therapies in the future.

## The Role of LDHA in Tumor Evasion of the Immune Response

It has been reported that a poor lymphocyte response occurring at the invading tumor edge is associated with carcinomas expressing high levels of LDHA 36. In addition, low lymphocyte response was associated with 17 out 20 non‐Hodgkin's lymphoma patients who had a medium or high LDHA expression 78. Again, it is thought that lactate generation, promoted by LDHA, is the predominant cause of LDHA‐mediated inflammation 117 and evasion of the immune response 45 by inhibiting monocyte migration 37 and differentiation to dendritic cells 38 and by inhibiting cytotoxic T cell and dendritic cell cytokine release 30. Like tumor cells, activated T cells are highly proliferative and use glycolysis as their primary energy source. However, in the high lactate environment surrounding the tumor, activated T cells cannot secrete their own lactate, which depends on the intra‐ to extracellular concentration gradient of lactate 30, 152. Conversely, regulatory T cells do not use glycolysis as their primary energy source and are not affected by the high lactate concentrations 84.

In brain tumors, a study in GBMs revealed that LDHA, secreted by the tumor, induced the transcription and expression of natural killer group 2 member D (NKG2D) ligands, ULBP‐1 (UL16 binding protein 1) and MICB [major histocompatibility complex (MHC) class I chain‐related B)], on circulating monocytes and tumor infiltrating myeloid cells 18. Chronic exposure to NKG2D ligands expressed by monocytes downregulates the expression of NKG2D receptors on natural killer cells, preventing their ability to lyse NKG2D ligand‐expressing tumor cells 93. Previous studies in glioma have shown that TGF‐β can also decrease NKG2D expression on NK cells *in vitro* 17. As discussed previously, lactate production by LDHA activates TGF‐β in glioma 6; therefore, it is possible that LDHA also activates TGF‐β to promote evasion of the immune response. This phenomenon should be investigated further.

## LDHA and the Tumor Microenvironment

Because of excessive tumor growth, the tumor microenvironment is often hypoxic and acidic which can effect protein expression. Oxygen levels often vary within a tumor, for example, GBMs have oxygenated areas around the tumor vasculature, as well as areas of oxygen‐deprived cells and necrosis. GBMs are well known to display intratumor heterogeneity which is caused, in part, by the microenvironment and also makes them more adaptable to small fluctuations within the microenvironment. As mentioned previously, hypoxia promotes transcription of LDHA by HIF‐1 and upregulation of LDHA causes lactate production, decreasing pH levels. Sørensen *et al* conducted experiments to closely examine LDHA mRNA expression in response to various oxygen and extracellular pH (pHe) conditions. They found that, in a human cervix squamous cell carcinoma cell line, after 24 h, LDHA mRNA levels increased slightly as oxygen decreased from 21% to 0.01% at pHe 7.5, 7.0 and 6.7. Furthermore, at pHe 6.5 and 6.3, and 1% oxygen, LDHA mRNA levels were six‐ to sevenfold higher than at 21% oxygen 118, 119. When they repeated the experiment in a pharyngeal squamous cell carcinoma cell line, they found that LDHA mRNA expression also gradually increased as oxygen concentrations decreased from 21% to 0.01%; however, changes in pHe had little effect on LDHA mRNA expression 119. These results indicate that LDHA expression in response to the tumor microenvironment is regulated differently in different tumors, which could be linked to the aggressiveness of the tumor or metabolic phenotype.

LDHA can influence the tumor microenvironment through generation of lactate which lowers extracellular pH. Tumor pH can also be variable within a tumor; using pH‐sensitive electrodes, primary brain tumors have been found to have a mean pH of 6.8 and as low as 5.9 compared with normal brain tissue which has a pH of 7.1 132. It has also been shown that an acidic pH induces glioma stem cell markers and promotes angiogenesis and malignancy; furthermore, *in vitro* elevation of pH reversed these effects 46. It is conceivable therefore that LDHA is a major manipulator of the tumor microenvironment, via lactate production and decreasing the environmental pH, which promotes a cancer stem cell phenotype, angiogenesis, migration and immune evasion.

## LDHA as a Therapeutic Target

**Figure 5:**
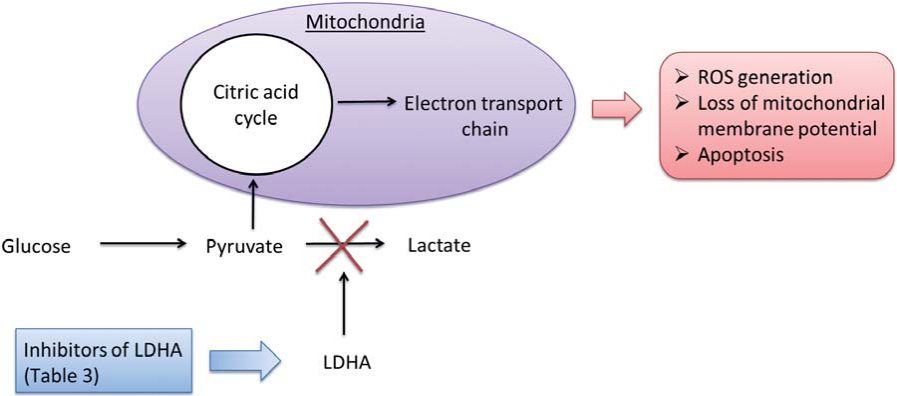
Schematic showing the reported mechanism in which lactate dehydrogenase A (LDHA) inhibitors cause apoptosis in cancer cells. Reported inhibitors of LDHA (listed in Table 3) obstruct aerobic glycolysis and the processes listed in Figure 3. Cancer cells are then forced to use oxidative phosphorylation and pyruvate enters the mitochondria. This leads to reactive oxygen species (ROS) generation and apoptosis.

As discussed previously, LDHA is associated with tumor initiation, maintenance, progression and poor prognosis in many tumors. High serum LDH concentrations are also associated with radio‐resistance in both primary and metastatic brain tumors. Moreover, multiple studies on various cell lines have shown that attenuation of LDHA increases apoptosis 27, 72 (Figure 5) and reduces migration and invasion ability 113, 140 demonstrating its use as a potential therapeutic target. Mouse model studies have found that LDHA deletion is embryonic‐lethal; however, when LDHA is switched off in the Cre^tm^–LDHA^fl/fl^ mouse model treated with tamoxifen, mice develop severe, nonlethal hemolytic anemia 141. Furthermore, human genetic defects in the LDHA gene are also nonlethal but do cause glycogen storage disease type 11 (GSD11). Together, these studies suggest that LDHA inhibition could be a well‐tolerated therapy that will impede tumor growth and metastasis.

To demonstrate the possible benefits of targeting LDHA as a therapeutic target, as well as using LDHA‐targeted siRNA and shRNA, many studies have also used oxamate, an analog of pyruvate that prevents LDH converting pyruvate to lactate and has been shown to work synergistically with other current therapies. Zhou *et al* showed that human breast cancer cells, which had become resistant to the chemotherapeutic agent Taxol, had increased levels of LDHA expression when compared to their parental cells 152. Furthermore, they found that downregulation of LDHA by using both LDHA‐targeted siRNA and oxamate increased the sensitivity of the Taxol‐resistant cells to Taxol and promoted apoptosis 152.

Miskimins *et al* conducted studies with oxamate and phenformin on six different cancer cell lines. Phenformin inhibits complex I in the mitochondria electron transport chain, causing excess ROS production and is also associated with high incidence of lactic acidosis; however, oxamate reduced the lactic acidosis side effect, and furthermore, it had a synergistic anti‐cancer effect when treated in combination with phenformin, reducing tumor size, glucose uptake, ATP generation and increasing tumor apoptosis *in vivo* 86. Oxamate also increased the sensitivity of nasopharyngeal cancer cell lines to ionizing radiation, synergistically enhancing apoptosis and suppressed the growth of tumor xenografts 144.

One of the problems with oxamate is that it has limited cell penetration; therefore, relatively high doses are required to have any significant effect. However, Galloflavin, a synthesized LDHA and LDHB inhibitor, has high levels of cell penetration. Galloflavin inhibited the growth of well‐differentiated (MCF‐7), chemotherapy‐resistant (MCF‐Tam) and aggressive triple negative (MDA‐MB‐231) breast cancer cell lines, despite their metabolic differences, with their mean IC_50_ around 125 μm 28. However, this is still a higher concentration than commonly used in chemotherapeutics. Preliminary toxicity data are, however, promising, as 400 mg/kg body weight injected i.p. into mice was not lethal 81. Furthermore, the highest tested dose of Galloflavin (250 μm) caused <30% reduction of ATP and lactate production of normal human lymphocytes and lymphoblasts but had a mild effect on their growth and survival 28.

Gossypol, a derivative of cotton seed oil, inhibits LDHA and LDHC and is highly soluble at physiological pH 73. Furthermore, it has been shown to have cytotoxic effects against a range of tumors, including glioma cell lines 16. Coyle *et al* found that gossypol treatment of mouse xenograft models decreased the mean weight of tumors by more than 50%, and furthermore, the most sensitive glioma cell lines had higher LDHA expression levels 16. Moreover, gossypol has been shown to be well tolerated in clinical trials and has also shown promise in recurrent malignant glioma trials 12, 15, 31, 120. Two more clinical trials with gossypol and GBM have been completed (NCT00540722 and NCT00390403) but the results are currently unavailable. FX11 [3‐dihydroxy‐6‐methyl‐7‐(phenylmethyl)‐4‐propylnaphthalene‐1‐carboxylic acid] is a promising new gossypol‐derived specific LDHA small molecule inhibitor that has recently become commercially available. Studies on human lymphoma and pancreatic cancer xenografts have shown that FX11 inhibited tumor progression and induced significant oxidative stress and necrosis 72.

Recently, over 900 plant extracts commonly used in medicine have been screened and their ability to inhibit LDHA was evaluated. Forty‐six herbs, including cinnamon, pink rose buds/petals, rhodiola root, witch hazel root and wintergreen, significantly inhibited LDHA, with IC_50_s <0.07 mg/mL. However, Chinese gallnut (*Melaphis chinensis gallnut*), bladderwrack (*Fucus vesiculosus*), kelp (*Laminaria japonica*) and babul (*Acacia arabica*) had the lowest IC_50_s of less than 0.001 mg/mL 23. Further investigations are required to identify the active compounds within these plant extracts which could be used in future therapies. High‐throughput screening also identified quinoline 3‐sulfonamides as NADH‐competitive LDHA inhibitors. Lead compounds had a 10‐ to 80‐fold selectivity for LDHA over LDHB and IC_50_s were 2–10 nm, but regrettably the use of quinolone 3‐sulfonamides is unacceptable *in vivo* because of their pharmacokinetic properties; having a low *in vivo* clearance and being incompatible with oral bioavailability 8.

Other groups have designed and synthesized specific LDHA inhibitor compounds. For example, Granchi *et al* developed N‐hydroxyindole‐based (NHI) inhibitors of LDHA, which they showed to inhibit pancreatic ductal adenocarcinoma growth, particularly under hypoxic conditions 40 and work synergistically with gemcitabine (first‐line standard treatment) 80. They also found that the novel NHI LDHA inhibitors significantly reduced MMP‐2 and MMP‐9 expression under hypoxia which they hypothesize to be the cause of the attenuated cell migration and invasion observed 80. Moorhouse *etal* designed novel bifunctional ligands that inhibit LDHA, by synthesizing “NADH‐like” and “substrate‐like” azido and alkyne functionalized fragments and linking them together to form 31 novel compounds. The compound that showed the most promise for further development had a relatively low IC_50_ of 14.8 mM, and a ninefold better activity against LDHA than sodium oxamate 87. Another group found that Mn(II) complexes, good functional mimics of catalase, inhibit LDHA and also decreased HIF1α expression in the HepG‐2 cell line 143. Together, these studies show that designing small drug‐like molecules to target LDHA is achievable and able to hinder tumor growth and maintenance but many still require further development to improve their specificity and potency.

Targeting LDHA and tumor metabolism downstream of pyruvate generation is an attractive option for cancer therapies as the effect on the metabolism of normal cells should be minimal. Although studies have clearly demonstrated the therapeutic potential of inhibiting LDHA in cancer patients, most current inhibitors (Table 3) affect more than one of the LDH genes, have low potency and require unacceptably high doses (which may also be affecting other unknown nonspecific targets) to achieve the desired effect and therefore are not suitable for clinical use. As described earlier, many groups are in the early stages of identifying and designing compounds which could eventually be used as LDHA inhibitors in clinical trials; however, it is of note that most novel LDHA inhibitors appear to be focused on targeting the LDHA enzyme activity rather than the protein expression itself. Nonetheless, studies showing synergistic effects of LDHA inhibitors in combination with other therapeutics such as radiation, phenformin, taxol and gemcitabine are promising. Although a few studies of LDHA in brain tumors have shown promise, the extent of these studies is severely lacking. Brain tumors are often more difficult to treat than other cancers as therapeutic drugs often have limited propensity to cross the protective BBB. However, to our knowledge, no groups have tested whether any potential LDHA inhibitors are able to cross the BBB.

**Table 3:** List of reported lactate dehydrogenase A (LDHA) inhibitors

LDHA inhibitor	Reference
Oxamate	86, 144, 152
FX11	72
Galloflavin	28, 81
Gossypol	12, 15, 16, 31, 120
Quinoline 3‐sulfonamides	8
N‐hydroxyindole‐based (NHI) inhibitors	40
Bifunctional ligands	87
Mn(II) complexes	143

## Conclusions

LDHA is clearly more than just a metabolic gene that is overexpressed in cancer and the true extent of its function and the exact mechanism in which it operates in non‐neoplastic and neoplastic cells is only just beginning to come to light. Extensive studies have shown that LDHA is involved directly and indirectly in many aspects of tumor growth, migration, invasion and maintenance in a wide range of tumors (Figure 3). Despite the promising findings of a few studies presented here, the significance of LDHA in normal brain function and brain tumor initiation and progression is an area of research that is not receiving enough attention. More research conducted on the less studied roles of LDHA should also be encouraged as findings will, no doubt, benefit the ongoing search for suitable LDHA inhibitors which could be used in future clinical trials. It is also imperative to keep the issue of crossing the BBB in mind when designing LDHA inhibitors.
